# Supplementary material for: Molecular movie of ultrafast coherent rotational dynamics of OCS
Source: Nat Commun. 2019 Jul 29;10:3364. doi: 10.1038/s41467-019-11122-y (PMC6662765; doi:10.1038/s41467-019-11122-y)
Supplement: Supplementary file 1 — Supplementary information [file 41467_2019_11122_MOESM1_ESM.pdf]

Molecular movie of ultrafast coherent rotational dynamics of OCS

Karamatskos et al.

Supplementary information: Molecular movie of ultrafast coherent rotational dynamics of OCS

Evangelos T. Karamatskos,[1,2] Sebastian Raabe,[3] Terry Mullins,[1]
Andrea Trabattoni,[1,2] Philipp Stammer,[3] Gildas Goldsztejn,[3] Rasmus R. Johansen,[4]
Karol Długołęcki,[1] Henrik Stapelfeldt,[4] Marc J. J. Vrakking,[3]
Sebastian Trippel,[1,5] Arnaud Rouzée,[3,‖] and Jochen Küpper[1,2,5,*]

[1] *Center for Free-Electron Laser Science, Deutsches Elektronen-Synchrotron DESY, Notkestraße 85, 22607 Hamburg, Germany*

[2] *Department of Physics, Universität Hamburg, Luruper Chaussee 149, 22761 Hamburg, Germany*

[3] *Max Born Institute, Max-Born-Straße 2a, 12489 Berlin, Germany*

[4] *Department of Chemistry, Aarhus University, Langelandsgade 140, 8000 Aarhus C, Denmark*

[5] *The Hamburg Center for Ultrafast Imaging, Universität Hamburg, Luruper Chaussee 149, 22761 Hamburg, Germany*

(Dated: June 12, 2019)

SUPPLEMENTARY NOTE 1: OPTIMISATION OF TWO-PULSE FIELD-FREE ALIGNMENT

Optimisation calculations were performed in order to predict the optimal pulse parameter for single and two-pulse field-free alignment. In the simulations, the rotational part of the Schrödinger equation for a linear rigid rotor within the Born-Oppenheimer approximation coupled to non-resonant ac alignment laser pulses and a static electric field, as provided by the VMI in the interaction region, was used. The Hamiltonian of the system is described in detail in reference [1]. The global differential-evolution-optimisation algorithm [2] was used to calculate the optimal alignment characterised through the expectation value $\langle\cos^2\theta\rangle$ in a closed-feedback-loop approach. The optimisation parameters used were the intensities and one common duration of Fourier-limited Gaussian pulses and the delay between the pulses in the case of two-pulse alignment. In the calculations a pure rotational ground state ensemble was assumed and no integration over the interaction volume was carried out. The former is justified as we know that the ground state contribution to alignment is dominant and the exact experimental conditions were not known a priori. Furthermore, exploiting the electrostatic deflector, as in our experiment, almost pure ground state ensembles can be prepared [3, 4]. Including also thermally excited rotational states lead to an additional incoherent sum over all states present in the initial distribution of rotational states and in general to a decrease of the degree of alignment. The same holds for the interaction volume of the laser since only molecules at the center of the beam experience the optimal alignment intensity while molecules at some distance from the center interact with a lower field. In this sense the calculated values constitute an upper limit for the alignment under optimal conditions. Time-evolution curves of the optimal two-dimensional (2D) and three-dimensional (3D) alignment are shown in Supplementary Figure 1. The results for the single pulse optimisation yielded a pulse duration of 114.5 fs and a maximum intensity of 5.6 TW cm^{-2}. The corresponding maximum degree of field-free 3D alignment was found to be $\langle\cos^2\theta\rangle = 0.92$, which is in agreement with an upper bound of 0.92 derived previously [5]; this corresponds to a 2D degree of alignment of $\langle\cos^2\theta_{2D}\rangle = 0.95$.

The optimal parameters for the case of two alignment pulses were found to be a pulse duration of 273.4 fs, a pulse separation between the two pulses of 38.2 ps, and an intensity-ratio of $\sim$1 : 5 with the first pulse being weaker than the second one, in agreement with previous

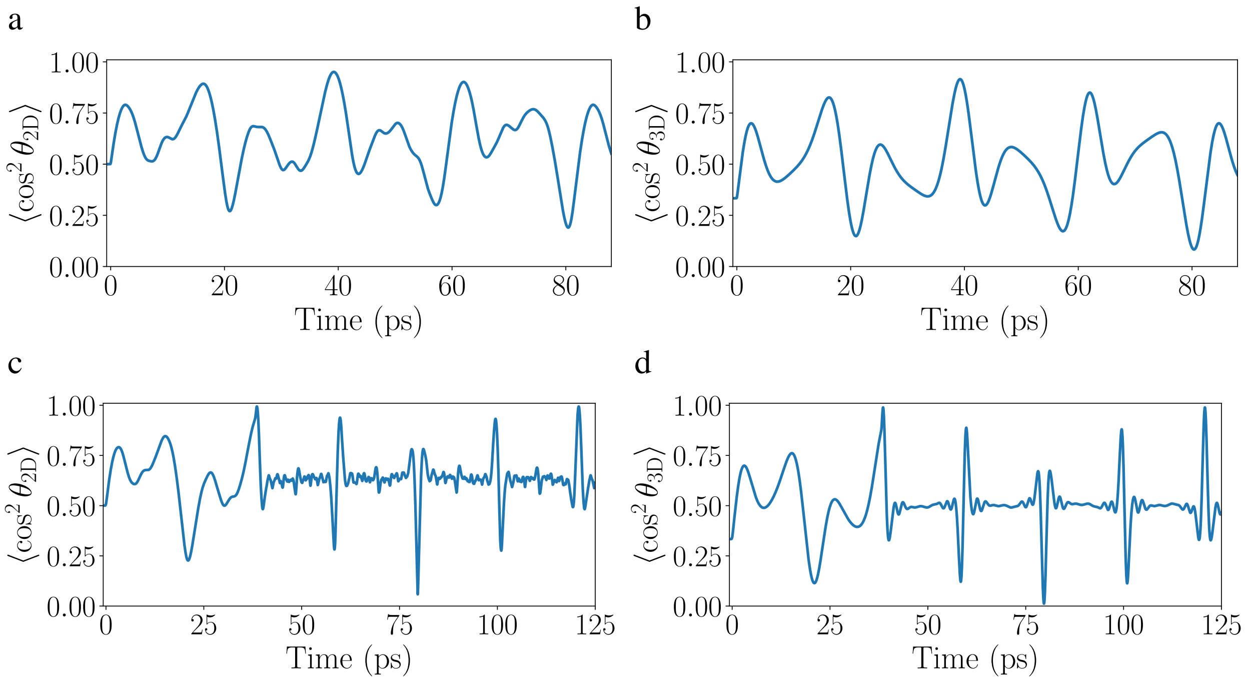

Supplementary Figure 1. **Simulations of optimised single- and two-pulse field-free alignment** Optimised **a, c** 2D and **b, d** 3D field-free alignment with **a, b** one and **c, d** two alignment pulses.

results [6, 7]. The maximum intensity of the first pulse was determined to be 1.93 TW cm^{-2} and that of the second pulse 10 TW cm^{-2}, which was the upper bound of intensities included in the calculations since for higher values at a wavelength of 800 nm a non-negligible amount of ionisation of OCS sets in. The maximum degree of 3D field-free alignment calculated with these parameters were $\langle\cos^2\theta\rangle = 0.99$ and $\langle\cos^2\theta_{2D}\rangle = 0.99$, substantially higher than in the single pulse case. The experiment presented in the main paper was performed under experimental conditions approximating these optimised parameter. We note that the optimal pulse separation was calculated to be 38.2 ps, which was confirmed in the experiment, for which a scan of the pulse separation yielded the best alignment revival for 38.1 ± 0.1 ps.

SUPPLEMENTARY NOTE 2: MOMENTS OF ANGULAR DISTRIBUTION

There are several ways to expand the angular distribution of the wavepacket in a power series, but a natural basis consists of the Legendre polynomials, as for $\Delta M = 0$ the eigenstates are independent of ϕ and the spherical harmonics simplify to Legendre polynomials. We

had indeed originally performed the analysis in terms of squared Chebyshev polynomials $\left\langle\cos^2 n\theta_{2D}\right\rangle$ for numerical convenience and the results of both approaches are identical. Only even order polynomials appear in the expansion since for a ground-state-selected ensemble the odd order moments describe orientation of the molecular axes, which was not present. The expansion takes on the form

$$P(\theta_{2D}, t) = \sum_{k=0, k\,\mathrm{even}}^{J_\mathrm{max}} a_k(t)\mathcal{P}_k(\cos\theta_{2D}) \tag{1}$$

where the full time-dependent angular distribution is denoted as $P(\theta_{2D}, t)$ and a_k ($k = 0, 2 \ldots J_\mathrm{max}$) are the expansion coefficients corresponding to the k-th Legendre polynomial $\mathcal{P}_k$; J_max is the angular momentum quantum number of the highest populated rotational state in the wavepacket.

In order to characterise the initial state distribution of rotational states in the molecular beam, the eight lowest even-order moments of the experimental angular distributions were fitted simultaneously using least squares minimisation. For each moment, squared differences were summed according to

$$\chi_n^2 = \sum_t \left(\langle\mathcal{P}_{2n}(\cos\theta_{2D})_\mathrm{exp}\rangle(t) - \langle\mathcal{P}_{2n}(\cos\theta_{2D})_\mathrm{sim,vol}\rangle(t)\right)^2 , \tag{2}$$

where the sum runs over all measured delay times t and $n = 1 \ldots 8$. In order to compute $\langle\mathcal{P}_n(\cos\theta_{2D})_\mathrm{sim,vol}\rangle(t)$, several steps were followed. First, the coherent wavepackets, created through the interaction with the alignment laser pulses, were for every initial state described in the basis of field-free eigenstates as

$$\Psi_{J_\mathrm{i},M_\mathrm{i}}(\theta, \phi, t) = \sum_J a_J(t) Y_J^{M_\mathrm{i}}(\theta, \phi), \qquad a_J(t=0) = \delta_{JJ_\mathrm{i}}, \tag{3}$$

where $a_J(t) = |a_J(t)|e^{i\phi_J(t)}$ are time-dependent complex coefficients with amplitude $|a_J(t)|$, phase $\phi_J(t)$, and initial condition $a_J(t=0) = \delta_{JJ_\mathrm{i}}$, δ_{JJ_i} is the Kronecker delta, obtained from the solution of the time-dependent Schrödinger equation; $Y_J^M(\theta, \phi)$ are the spherical-harmonic functions and $J_\mathrm{i}, M_\mathrm{i}$ are the quantum numbers of the initial state from which the wavepacket is formed. The sum runs only over J, since M was a good quantum number due to cylindrical symmetry, as imposed by the linear polarisation of the alignment laser, and, hence, $\Delta M = 0$ and $M = M_\mathrm{i}$ was conserved. Furthermore, the selection rules for transitions between different rotational states were $\Delta J = \pm 2$, since the population transfer is achieved via non-resonant

two-photon Raman transitions. Moreover, the static VMI field was perpendicular to the alignment laser polarisation and does not mix different M states. Since more than one rotational state were initially populated, the 3D rotational density was obtained through the incoherent average with statistical weights $w_{J_\mathrm{i},M_\mathrm{i}}$

$$P_{\mathrm{sim,3D}}(\theta,\phi,t) = \sum_{J_\mathrm{i},M_\mathrm{i}} w_{J_\mathrm{i},M_\mathrm{i}}\, p(\theta)\left|\Psi_{J_\mathrm{i},M_\mathrm{i}}(\theta,\phi,t)\right|^2 , \tag{4}$$

which were not known *a priori* and used as fitting parameters. The function $p(\theta)$ describes the angle-dependent ionisation probability, which was approximated through the square of the measured angular-dependent single-electron ionisation rate. Finally, a focal average over the interaction region with the alignment and probe laser beam profiles, assumed to be Gaussian, was performed. The average over intensities in the laser focus was calculated through integration

$$\left\langle P_{\mathrm{sim,3D}}(\theta,\phi,t)\right\rangle_{\mathrm{vol}}(t) = \frac{1}{N}\int_0^{r_{\mathrm{max}}} \left\langle P_{\mathrm{sim,3D}}(\theta,\phi,I_{\mathrm{align}}(r),t)\right\rangle e^{-2r^2/w_{\mathrm{probe}}^2}\, r\,\mathrm{d}r \tag{5}$$

with radius r_{max} at $I_{\mathrm{align}} = 10^9$ W cm^{-2} and N a normalisation factor. The dependence of the rotational wavepackets on the alignment laser intensities is explicitly stated in (5). The widths of the laser beams were also not known *a priori* and were included as further fitting parameters. The resulting focal- and initial-state-averaged 3D rotational densities were projected onto a 2D plane using a Monte-Carlo sampling routine, which included the experimental radial distribution extracted at the full revival at a delay time of 120.78 ps, yielding the simulated VMI images in Fig. 2 in the main paper. The relation between the 3D rotational density and the 2D projected density is graphically illustrated in Supplementary Figure 2. The Legendre moments of the angular distribution were then extracted from the 2D projected images and compared to experiment through χ_n^2, as described in (2). The statistical weights $w_{J_\mathrm{i}M_\mathrm{i}}$ of the initial state distribution and the laser focal sizes were varied until $\sum_n \chi_n^2$ converged to its minimum. The individual populations determined through the fitting procedure are $w_{00} = 0.82(3)$, $w_{10} = 0.037(3)$, $w_{11} = 0.075(3)$, $w_{20} = 0.015(2)$, $w_{21} = 0.021(3)$, and $w_{22} = 0.032(3)$. The optimal focal parameter were determined to be $w_{\mathrm{align}} = 130(15)$ µm for the alignment laser and $w_{\mathrm{probe}} = 60(7)$ µm for the probe laser. Numbers in parentheses are 1σ standard deviations in the last digits, which were estimated from the least squares minimisation using the covariance matrix. The results are consistent

with the fact that the probe laser was focused tighter than the alignment laser such that only molecules exhibiting strong alignment, close to the beam centre, were probed.

The final results of the fitting procedure are shown in Fig. 2 in the main paper and in Supplementary Figure 3. The simulated angular distributions and the moments of the angular distribution are in excellent agreement with the experiment, in particular all oscillations are correctly captured, even for the highest-order moments. The experimental parameters used for the simulations were the peak intensities for the two alignment pulses of $I_{\text{align},1} = 1.92$ TW cm^{-2} and $I_{\text{align},2} = 5.5$ TW cm^{-2}, the pulse duration of the alignment laser pulses $\tau_{\text{align}} = 255$ fs, the time delay between the two alignment laser pulses $\tau_{\text{delay}} = 38.1$ ps, and the pulse duration of the probe laser $\tau_{\text{probe}} = 60$ fs. Calculations with 21 initial states, that is $J = 0 \ldots 5, M = 0 \ldots 5$, included in the initial rotational state distribution were originally performed, but convergence was already reached for the 6 lowest-energy states and the fitting procedure was restricted to using these 6 lowest rotational states, that is $J = 0 \ldots 2, M = 0 \ldots 2$, and the focal volume was averaged over 100 intensities in $I_{\text{align}} = 1 \cdot 10^9 \ldots 5.5 \cdot 10^{12}$ W cm^{-2}. In all calculations the basis for each coherent wavepacket included all rotational states up to $J = 50$.

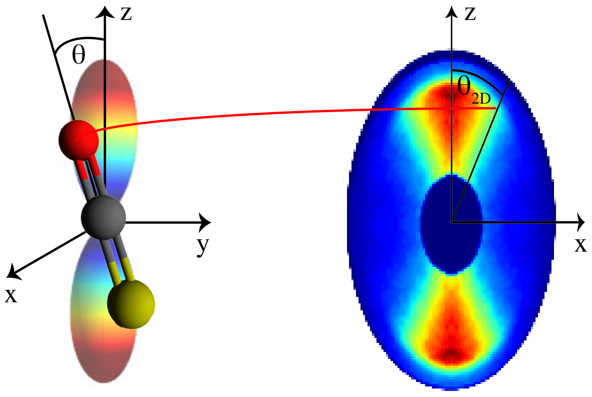

Supplementary Figure 2. **Sketch of 2D projection of rotational density, showing the relation between θ and θ_{2D}** Relation between the Euler angle θ, defining the alignment of the molecular axis with respect to the pump laser polarisation axis, and θ_{2D}, corresponding to the angle between the pump laser polarization and the detected ion-momentum distribution on the 2D detector.

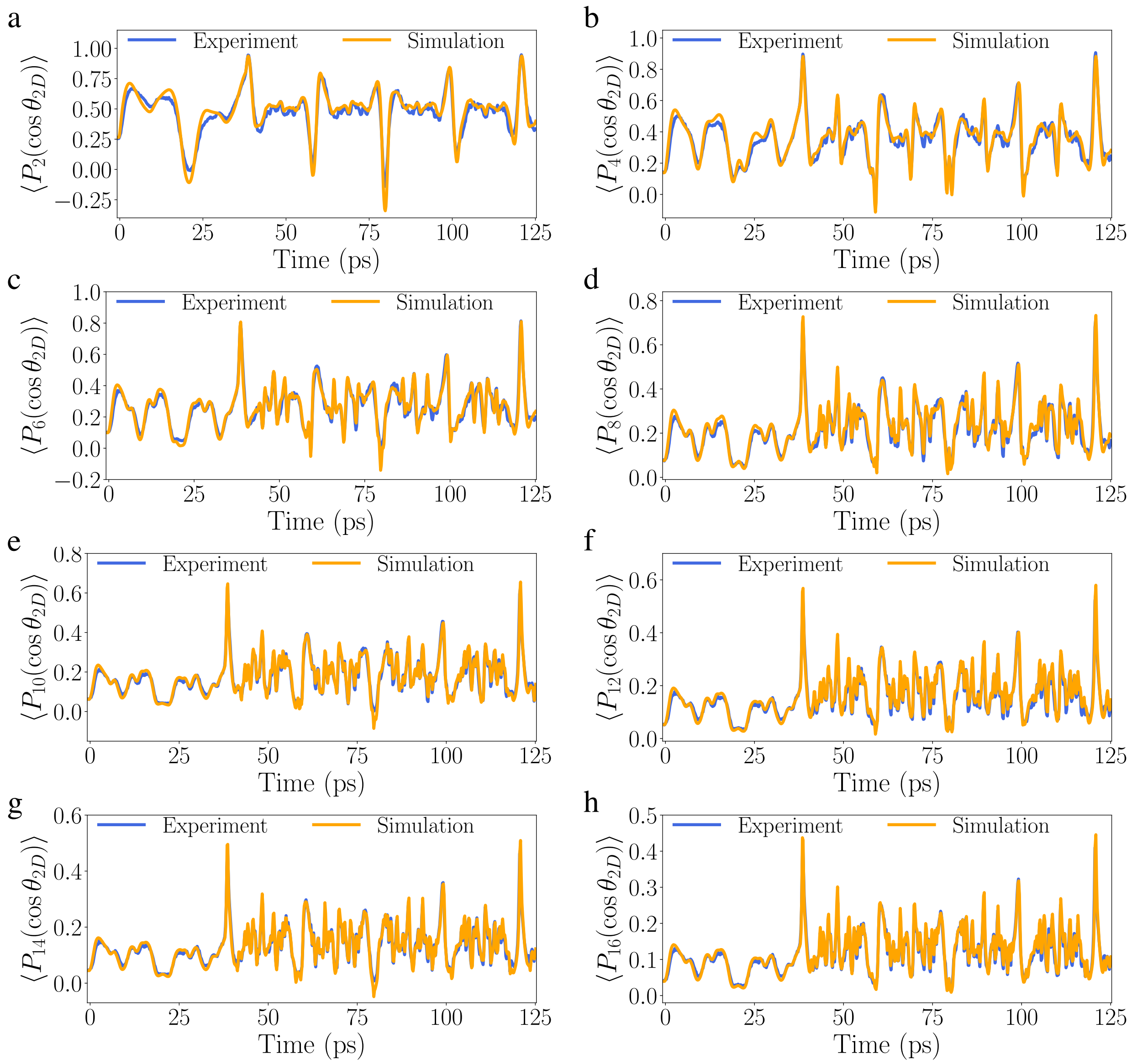

Supplementary Figure 3. **Even order moments of angular distributions expanded in Legendre polynomials** Even order moments 1 to 8 of the angular distribution **a** $\langle P_2(\cos\theta_{2D})\rangle$, **b** $\langle P_4(\cos\theta_{2D})\rangle$, **c** $\langle P_6(\cos\theta_{2D})\rangle$, **d** $\langle P_8(\cos\theta_{2D})\rangle$ **e** $\langle P_{10}(\cos\theta_{2D})\rangle$, **f** $\langle P_{12}(\cos\theta_{2D})\rangle$, **g** $\langle P_{14}(\cos\theta_{2D})\rangle$, **h** $\langle P_{16}(\cos\theta_{2D})\rangle$.

SUPPLEMENTARY NOTE 3: ANGULAR DISTRIBUTIONS

As pointed out in the main text, we observed angular probability distributions showing the very rich time evolution of the rotational wavepacket created by the two alignment laser pulses. When characterising the degree of alignment using the commonly used $\langle \cos^2\theta_{\rm 2D}\rangle$ we

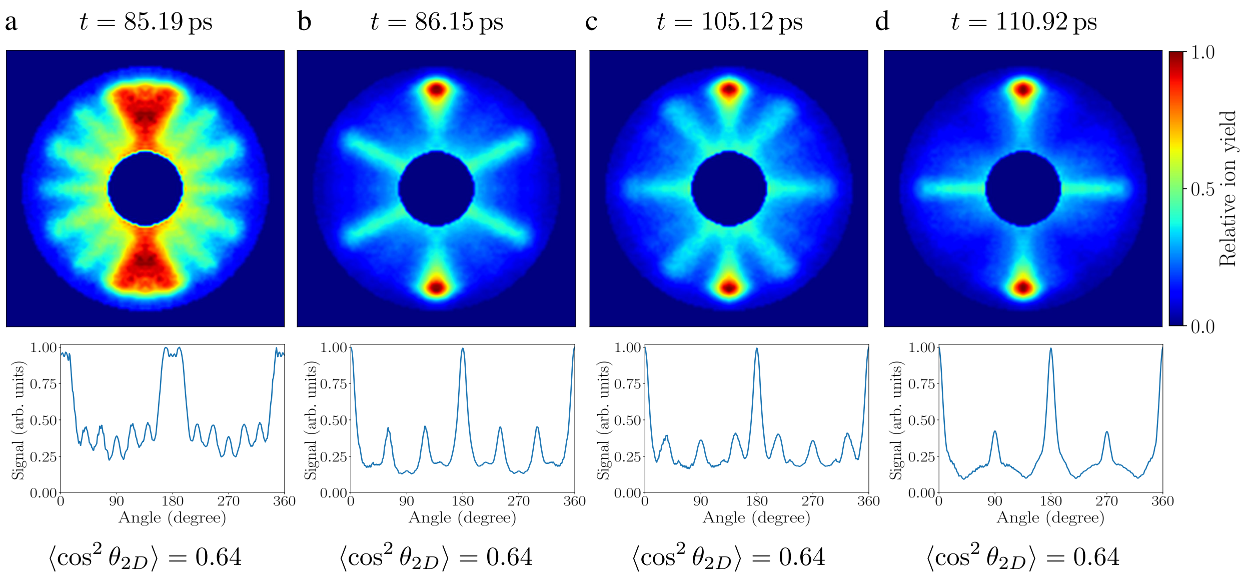

Supplementary Figure 4. **O^+ ion momentum probability distributions** These four distributions were recorded at different time delays, displaying very different angular distributions, but having all the same degree of alignment of $\left\langle \cos^2\theta_{2D} \right\rangle = 0.64$. These distributions were recorded for delay times of **a** 85.19 ps, **b** 86.15 ps, **c** 105.12 ps, and **d** 110.92 ps; cf. [8, Fig. 10].

observed that completely different angular distributions possess the same degree of alignment, which pointed out the need for higher order terms in the expansion of the total angular distribution, e. g., in the basis of Legendre polynomials, to be able to unravel the complete rotational wavepacket. In Supplementary Figure 4 we present O^+ ion momentum distributions measured at four different delay times together with their corresponding angular distributions corroborating this observation. The delay times were chosen such that all distributions have the same $\left\langle \cos^2\theta_{2D} \right\rangle = 0.64$, corresponding to the permanent alignment level. Although the degree of alignment is quite low compared to the maximum degree of alignment achieved, one clearly sees in particular in Supplementary Figure 4 b–d that nevertheless there is a substantial amount of molecules being strongly aligned. Thus it is clearly not sufficient to just use the degree of alignment in terms of $\left\langle \cos^2\theta_{2D} \right\rangle$ to characterise the molecular alignment distribution, but the knowledge of the whole angular distribution is needed.

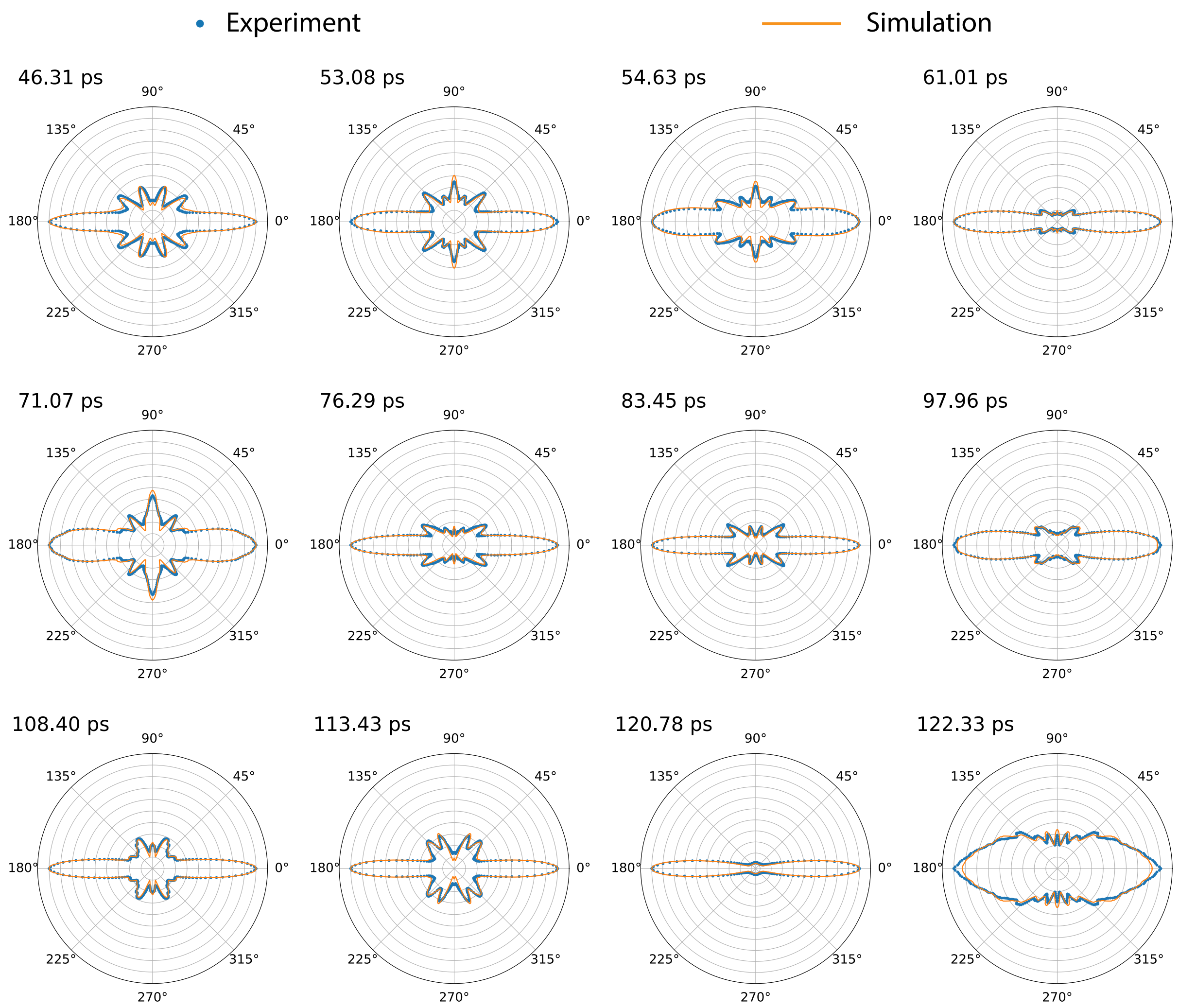

Supplementary Figure 5. **Comparison of experimental and simulated angular distributions**, shown for some selected delay times after the second alignment laser pulse has arrived.

Comparison of angular distributions from experiment and simulations

We show a comparison of angular distributions extracted from experiment and from the simulated, 2D projected rotational densities for selected times, starting after the arrival of the second alignment laser pulse in Supplementary Figure 5. The angular distributions display rich features, the simplest one being the alignment revival at a delay time of 120.78 ps. For better visibility the angular distributions have all been scaled up individually to maximise visibility, except for the alignment revival at 120.78 ps.

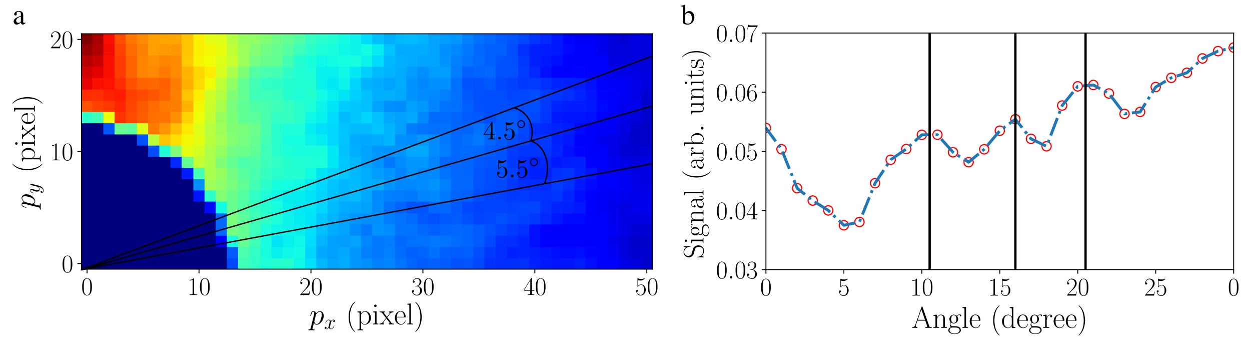

Supplementary Figure 6. **Determination of angular resolution a** Part of the recorded VMI image at a delay time of 98.5 ps is shown with lines indicating the angles at which maxima in the angular distribution were observed. **b** Angular distribution for the same cutout of the VMI image, shown with the position of the maxima indicated by vertical lines. The smallest measured angle between maxima in the angular distribution is 4.5 °, close to the angular resolution of 4 °.

SUPPLEMENTARY NOTE 4: ANGULAR RESOLUTION

The angular resolution was defined by the radius of the Coulomb channel in the VMI image, at which the angular distribution was extracted, and the number of pixels needed to distinguish two successive maxima or minima. The center of the radial Coulomb channel was at a radius of 46 pixel, which yielded an angle of 1.26 ° per pixel, corresponding to a limit for the resolution to separate two maxima or minima of 4 °. In Supplementary Figure 6 a, a O^+ ion momentum distribution recorded at a delay time of 98.5 ps is shown with lines indicating the angles at which maxima in the angular distribution appear. In Supplementary Figure 6 b the corresponding angular distribution is shown, where the maxima are clearly visible and distinguishable.

SUPPLEMENTARY NOTE 5: HIGHEST OBSERVED DEGREE OF ALIGNMENT

In Supplementary Figure 7 a the O^+ ion momentum distribution of the strongest observed field-free alignment is shown. The image was recorded at a delay time of 120.78 ps, which is the alignment revival, one rotational period after the arrival of the second alignment pulse. The degree of alignment was $\langle\cos^2\theta_{2D}\rangle = 0.96$ as stated in the main text. The value was obtained through integration in the shell between $r_{min} = 40$ and $r_{max} = 64$. In Supplementary

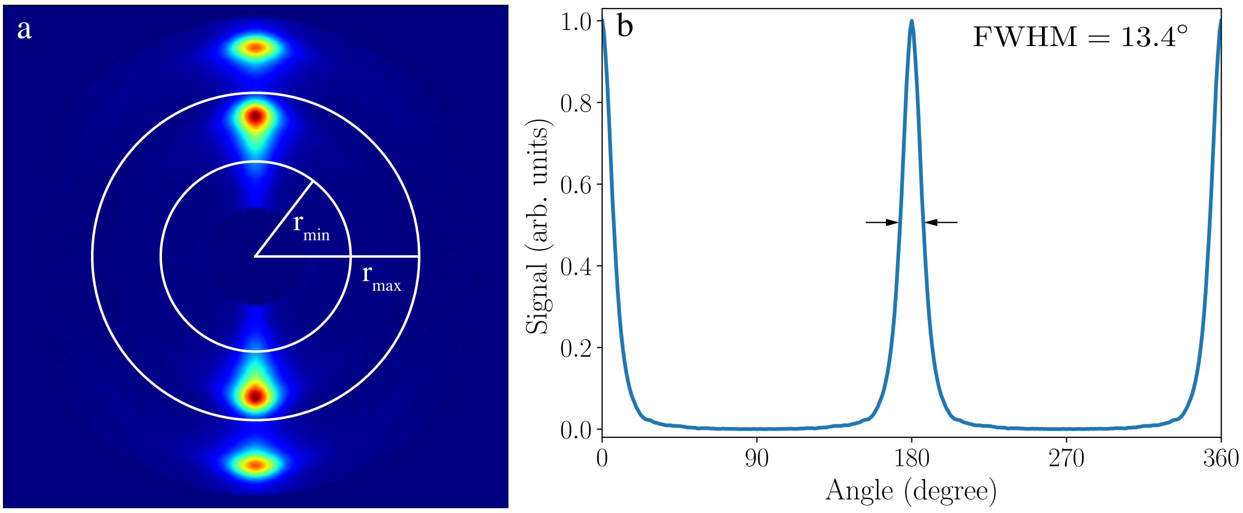

Supplementary Figure 7. **Observed highest degree of alignment of** $\langle\cos^2\theta_{\mathbf{2D}}\rangle = 0.955$ **a** O^+ ion momentum distribution recorded at the alignment revival at a delay time of 120.78 ps. The integration for the calculation of $\langle\cos^2\theta_{2D}\rangle$ was carried out in the shell between $r_{\text{min}} = 40$ and $r_{\text{max}} = 64$ pixel. **b** Corresponding angular distribution with a full opening angle of FWHM = 13.4°

Figure 7 b the corresponding angular distribution is shown, which yielded an opening angle of FWHM = 13.4°.

SUPPLEMENTARY NOTE 6: MOVIES

We provide two MP4, Supplementary Movie 1 and Supplementary Movie 2, movie files that show the complete measured and computed time-dependent angular probability densities corresponding to Fig. 1 in the main manuscript:

- `OCS alignment movie - experimental.mp4`
- `OCS alignment movie - computed.mp4`

SUPPLEMENTARY REFERENCES

‖ arnaud.rouzee@mbi.de

* jochen.kuepper@cfel.de; https://www.controlled-molecule-imaging.org

[1] J. J. Omiste and R. González-Férez, "Nonadiabatic effects in long-pulse mixed-field orientation of a linear polar molecule," Phys. Rev. A **86**, 043437 (2012).

[2] R. Storn and K. Price, "Differential evolution – a simple and efficient heuristic for global optimization over continuous spaces," J. Glob. Opt. **11**, 341–359 (1997).

[3] J. H. Nielsen, P. Simesen, C. Z. Bisgaard, H. Stapelfeldt, F. Filsinger, B. Friedrich, G. Meijer, and J. Küpper, "Stark-selected beam of ground-state OCS molecules characterized by revivals of impulsive alignment," Phys. Chem. Chem. Phys. **13**, 18971–18975 (2011), arXiv:1105.2413 [physics].

[4] Y.-P. Chang, D. A. Horke, S. Trippel, and J. Küpper, "Spatially-controlled complex molecules and their applications," Int. Rev. Phys. Chem. **34**, 557–590 (2015), arXiv:1505.05632 [physics].

[5] S. Guérin, A. Rouzée, and E. Hertz, "Ultimate field-free molecular alignment by combined adiabatic-impulsive field design," Phys. Rev. A **77**, 041404 (2008).

[6] C. Bisgaard, M. Poulsen, E. Péronne, S. Viftrup, and H. Stapelfeldt, "Observation of enhanced field-free molecular alignment by two laser pulses," Phys. Rev. Lett. **92**, 173004 (2004).

[7] M. Leibscher, I. Averbukh, and H. Rabitz, "Molecular alignment by trains of short laser pulses," Phys. Rev. Lett. **90**, 213001 (2003).

[8] P. W. Dooley, I. V. Litvinyuk, K. F. Lee, D. M. Rayner, M. Spanner, D. M. Villeneuve, and P. B. Corkum, "Direct imaging of rotational wave-packet dynamics of diatomic molecules," Phys. Rev. A **68**, 023406 (2003).
