# Supplementary material for: Molecular movie of ultrafast coherent rotational dynamics of OCS
Source: Nat Commun. 2019 Jul 29;10:3364. doi: 10.1038/s41467-019-11122-y (PMC6662765; doi:10.1038/s41467-019-11122-y)
Supplement: Supplementary file 5 — Description of Additional Supplementary Files [file 41467_2019_11122_MOESM5_ESM.docx]

**Description of Additional Supplementary Files**

File Name: Supplementary Movie 1

Description: Supplementary Movie 1 shows the complete measured movie of the time-dependent angular probability density, build up from individual raw experimental images of O^+^ momentum distributions. They reflect the time-evolution of the rotational wavepacket, created by the two alignment laser pulses, and constitute a direct visualisation of the interference between all

populated field-free rotational states.

File Name: Supplementary Movie 2

Description: Supplementary Movie 2 shows the complete simulated movie of the time-dependent angular probability density, build up from individual simulated images of O^+^ momentum distributions. They were computed by solving the time-dependent Schrödinger equation for the experimental parameters, including averages over the focal volume of the alignment and probe laser beams and the initial rotational-state distribution in the experiment. The computed angular-probability distributions were projected onto a 2D screen, thereby mimicking the experimental arrangement.
